# Nanoparticles Targeting Receptors on Breast Cancer for Efficient Delivery of Chemotherapeutics

**DOI:** 10.3390/biomedicines9020114

**Published:** 2021-01-26

**Authors:** Sulltana Jahan, Md. Emranul Karim, Ezharul Hoque Chowdhury

**Affiliations:** Jeffrey Cheah School of Medicine and Health Sciences, Monash University Malaysia, Jalan Lagoon Selatan, Bandar Sunway, Petaling Jaya 47500, Malaysia; romanakarim31@gmail.com (S.J.); karim604306@gmail.com (M.E.K.)

**Keywords:** breast cancer, nanoparticle, passive targeting, active targeting, chemotherapeutics, receptor targeted drug delivery, preclinical studies, therapeutic evaluation, translational gap

## Abstract

The journey of chemotherapeutic drugs from the site of administration to the site of action is confronted by several factors including low bioavailability, uneven distribution in major organs, limited accessibility of drug molecules to the distant tumor tissues, and lower therapeutic indexes. These unavoidable features of classical chemotherapeutics necessitate an additional high, repetitive dose of drugs to obtain maximum therapeutic responses with the result of unintended adverse side effects. An erratic tumor microenvironment, notable drawbacks of conventional chemotherapy, and multidrug-resistant mechanisms of breast cancer cells warrant precisely designed therapeutics for the treatment of cancers. In recent decades, nanoparticles have been deployed for the delivery of standard anticancer drugs to maximize the therapeutic potency while minimizing the adverse effects to increase the quality and span of life. Several organic and inorganic nanoplatforms that have been designed exploiting the distinctive features of the tumor microenvironment and tumor cells offer favorable physicochemical properties and pharmacokinetic profiles of a parent drug, with delivery of higher amounts of the drug to the pathological site and its controlled release, thereby improving the balance between its efficacy and toxicity. Advances to this front have included design and construction of targeted nanoparticles by conjugating homing devices like peptide, ligand, and Fab on the surface of nanomaterials to navigate nanoparticledrug complexes towards the target tumor cell with minimal destruction of healthy cells. Furthermore, actively targeting nanoparticles can facilitate the delivery and cellular uptake of nanoparticle-loaded drug constructs via binding with specific receptors expressed aberrantly on the surface of a tumor cell. Herein, we present an overview of the principle of targeted delivery approaches, exploiting drug-nanoparticle conjugates with multiple targeting moieties to target specific receptors of breast cancer cells and highlighting therapeutic evaluation in preclinical studies. We conclude that an understanding of the translational gap and challenges would show the possible future directions to foster the development of novel targeted nanotherapeutics.

## 1. Introduction

Globally, breast cancer is the most recurring malignancy and a foremost cause of cancer-related deaths in women, with the second highest incidence rate (11.6%) among all other cancers [1]. According to the WHO cancer profile 2020, 269,792 breast cancer cases were diagnosed in the year 2018 and 2,778,850 newly diagnosed cases are expected in 2040 (WHO 2020). There is an increasing rate of 3.19% of new cases recorded each year from 1980 to 2010 worldwide despite remarkable advances in cancer diagnosis and treatment [2]. This growing pattern warranted more concern about breast cancer diagnosis and management by medical scientists and health lawmakers [3]. Breast cancer is usually subdivided into two major classes: ductal and lobular carcinomas based on their histological constituents. Moreover, based on their molecular characteristics, like the expression of progesterone receptor (PR), estrogen receptor (ER), and human epidermal growth factor receptor 2 (HER2), breast cancers are further categorized into luminal A (ER+ PR+, and HER2-), luminal B (ER+/PR+ and HER2+/−), HER2 positive (ER−/PR− and HER2+), triple-negative/basal-like (ER−, PR−, and HER2−), and claudin-low [4,5,6]. Also, the American Joint Committee on Cancer (AJCC) defined eight stages of breast cancer based on tumor-node metastasis (TNM) [7,8]. Treatment modalities for breast cancer have been designed by taking into consideration the molecular portrait of heterogenicity, receptor expression, and stages of breast cancers to improve therapeutic performances. Early detected breast cancers are not lethal and curable in most of the cases (72–80%), while advanced-stage breast cancers are life-threatening and the leading cause of breast cancer-induced deaths owing to their metastasis to distant vital organs like the liver, lungs, brain, lymph nodes, and bone [9]. However, treatment has been designed aiming to increase life span with minimum unintended and undesirable side effects for improved quality of life. Current cancer treatment modalities are based on two major pillars, including localized treatment and systemic therapy. Surgical intervention is the first therapeutic option for early-stage nonmetastatic breast cancer patients. On the other hand, treatment options for locally advanced breast cancer include mastectomy or lumpectomy, chemotherapy, and high energy waves radiation therapy [10]. To date, systemic therapies including endocrine therapy, targeted therapy, and immunotherapy have been added based on the type and stage of breast cancer to get better therapeutic efficacy than the conventional ones. Although conventional treatment strategies are successful to some degree, their significant drawbacks like limited accessibility of a drug to distant tumor cells, higher therapeutic dose, nonspecific targeting, uneven biodistribution, and unwanted side effects, limit the efficiency of the therapy. Besides, multidrug resistance (MDR) of cancer cells against chemotherapeutic agents is another reason for chemotherapy failure. Chemoresistance may develop by modulating the influx or efflux of drugs, by activating survival pathways, or by augmenting DNA repair mechanisms to lessen the cytotoxic effects of the drug [1,11]. Therefore, the development of new modalities is desirable for cancer management that can specifically target cancer cells, thereby lowering the unwanted adverse effects with improved anticancer effects. In this regard, cancer nanomedicine has been introduced for the last few decades to promote the targeted delivery of chemotherapeutics to target tumor sites and to thus improve the therapeutic index. Nanoparticles (NPs) (10 to 400 nm) have added a new dimension in the drug delivery concept, providing an unprecedented opportunity to revolutionize cancer therapy and management. The immense potentiality of NPs as a drug carrier for specific identification and destruction of cancer cells led to an increased interest to study various biocompatible and biodegradable nanocarriers for the delivery of anticancer agents both in preclinical and clinical trials [12,13,14]. Considering the drawbacks of conventional therapies, NP-based drug delivery is a useful toolkit particularly for site-specific drug delivery, improved drug accumulation in tumor site due to the enhanced permeability and retention (EPR) effect, and the capability to bypass the MDR effects of the breast cancer cell [15,16,17,18]. In modern therapy, NPs can be further modified by equipping contrast agents, termed as theranostics for cancer diagnosis and treatment simultaneously [19]. In this review, we discuss different approaches to the targeted delivery of nanotherapeutics to cancer cells. We present a comprehensive review of the current applications and preclinical studies of various actively targeted nanoparticles for specific delivery of different chemotherapeutics agents used for the management of breast cancer. Finally, we shed light on the challenges and translational gap of receptor-targeted drug delivery and on the direction to future studies for its safer human use.

## 2. Insights of NP-Based Targeted Drug Delivery

**Figure 1:**
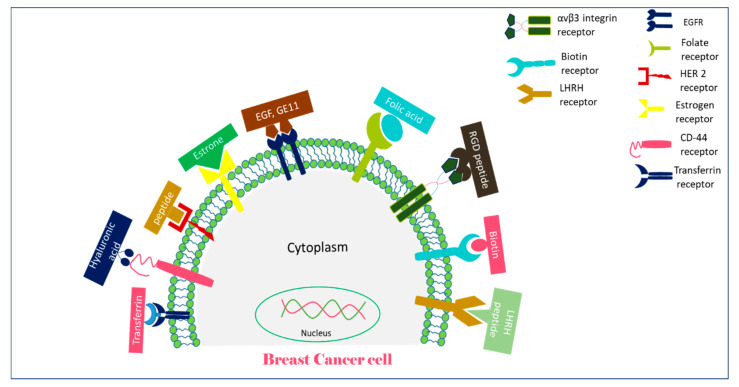
A schematic representation of receptors overexpressed on breast cancer cells and their targeting moieties for targeted breast cancer therapy.

**Figure 2:**
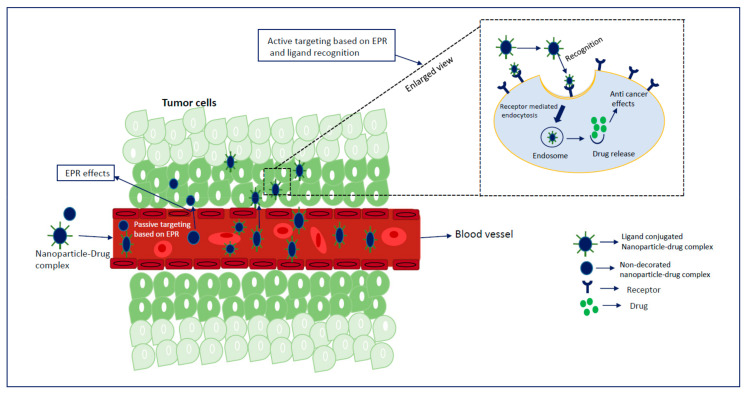
Combination strategy for drug targeting to a solid tumor using nanoparticles (NPs): after entering the systemic circulation, NPs are passively targeted and accumulated in the target tumor site by virtue of the enhanced permeability and retention (EPR) effect. Active targeting is achieved by decorating the surface of NPs with a targeting moiety and by capitalizing the advantages of aberrantly expressed receptors on the surface of tumor cells and EPR effects, resulting in improved drug delivery through receptor-mediated endocytosis.

**Figure 3:**
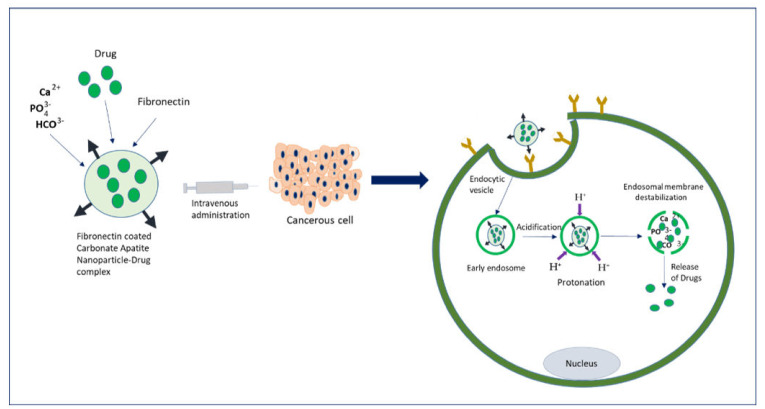
An overview of the drug delivery mechanism of fibronectin-coated pH-sensitive carbonate apatite (CA) NPs to the cancer cells expressing the fibronectin-specific integrin: upon systemic administration, drug-NP complexes are internalized into the target cell through specific integrin-mediated endocytosis. The acidic environment of an early endosome causes dissolution of the endocytosed NPs, resulting in their dissolution and release of the drugs, an event that might lead to endosomal membrane destabilization through accumulated ion-induced osmotic pressure, triggering quick release of drugs into the cytosol.

Extensive research on cancer therapeutics introduce several new paradigms to surmount conventional treatment drawbacks over the years. Upon systemic administration, chemotherapeutic drugs have to face several hurdles like premature degradation before reaching the target site, uneven biodistribution, and failure to discriminate healthy and cancer cells in addition to the requirement for their larger and repeated administration [20,21,22,23]. These barriers are considered responsible for severe unwanted side effects and high treatment costs of conventional chemotherapy [24,25]. Thus, a new way of delivering drugs specifically into a target tumor cell is a prerequisite to maximizing therapeutic efficacy and patient compliance. Emerging as a leading contender in the field of targeted drug delivery, NP-based therapeutics comprise therapeutic molecules such as small molecule drugs, peptides/proteins, and nucleic acids (genes and siRNAs) encapsulated into or associated with nanomaterials like liposomes [26,27], polymeric NPs [28,29], and inorganic NPs [30,31]. Since August 2014, 74 completed clinical trials and 57 trails with the status of “active” or “recruited” have been listed in clinicaltrials.gov, over 40,000 citations have been recorded in PubMed on NP-based drug delivery [21,32,33,34,35], and many more are on several stages of drug pipelines [17,36,37,38]. Preclinical studies showed NPs with loaded genes or drug-enhanced antitumor effects with an improved pharmacokinetic profile compared to free drugs [39,40,41] by virtue of the unique physicochemical properties of NPs, including loading capacity for both hydrophobic and hydrophilic drugs, ease of functionalization with targeting moieties and hydrophilic molecules, stability and extended half-lives in the systemic circulation, facilitated tumor accumulation due to their small particle size and EPR effect, enhanced cellular internalization via endocytosis, and quick and sustained release of the drugs in response to various intracellular (e.g., acidic pH) and extracellular (e.g., magnetic field, laser) stimuli [42,43,44]. Additionally, the size, conformation, and surface properties of NPs navigate the trajectory dynamics of the NP-loaded drug complex and are considered fundamental driving forces for improved delivery efficiency [45,46,47]. The passive targeting of NPs capitalizes on the anatomical variances of cancerous tissues. In solid tumors, rapid and abnormal angiogenesis results in an anomalous tumor microenvironment characterized by hyperpermeable tumor vasculature, poor lymphatic drainage, and unorganized extracellular matrix (ECM), allowing the nanocarriers to specifically penetrate through the fenestrate blood vessels and to be retained in the tumor tissue through the EPR effect [48,49,50]. The physicochemical properties and plasma half-life of NPs are considered the main factor for effective passive targeting, taking advantage of the EPR effect. A study showed that NPs with size range of 10–200 nm are enough to avoid clearance by the kidney and reticuloendothelial system (RES) and to facilitate maximum extravasation in the tumor site, whereas NPs with a surface charge slightly negative or neutral are considered ideal for avoiding protein corona effects [51,52,53,54,55]. However, passive targeting regulates tumor accumulation without triggering cellular entry, and a recent meta-study showed less than 1% of injected NPs accumulated in the targeted tumor site even with a high EPR effect in the xenografted tumor [56]. Besides, the idea of the EPR effect does not apply to all tumors. On the other hand, active targeting utilizes homing devices like ligands on the surface of NPs to let them target specifically for binding with specific receptors explicitly expressed on cancerous cells (Figure 1). The combined effects of EPR and active targeting might increase the retention of drug-loaded NPs at the target tumor site and thus promote cellular drug uptake through receptor-mediated endocytosis (Figure 2). In the last 10 years, more than 40,000 studies on active targeting of NPs have been reported, with few of them in the clinical trial [17,37,38]. A large number of targeting ligands including proteins, polysaccharide, nucleic acid, peptides, antibodies, and endogenous hormone have been employed for the construction of actively targeting NPs [57,58,59,60]. Usually, NPs can be functionalized with the targeting moiety by either chemical conjugation or a physical adsorption process. Modification of NPs with specific ligands targeting unique and overexpressed receptors or antigens on tumor cells is supposed to deliver the drug to the target tumor cells without hampering normal cells. Few studies reported that dual receptor-targeting strategies, multivalent binding, and use of a higher density of ligands are beneficial for maximizing binding affinity [61,62]. Despite having substantial successful preclinical studies, active targeting suffers from several limitations like poor penetration of the abnormal tumor microenvironment, relative hypoxia, an unknown endosomal escape mechanism, and relatively complex manufacturing steps for large-scale production. These shortcomings could be resolved by applying adjuvant therapy like vascular normalizing agents and other agents, such as TNF-α and angiotensin-II. Additionally, pH-responsive carbonate apatite (CA) (Figure 3) and strontium sulfite NPs could be employed to ensure quick release of the drugs from the NPs and subsequent endosomal escape of the free drugs at early endosomal stage [30]. A schematic diagram in Figure 3 shows how fibronectin-coated CA NPs could facilitate specific-integrin-targeted drug delivery to cancer cells.

## 3. Receptor-Targeted Delivery of Chemotherapeutic Agents in Mouse Breast Cancer Models

### 3.1. EGFR-Targeted Drug-Loaded NPs

As a transmembrane receptor, EGFR belongs to receptor tyrosine kinases (RTK) family, which is activated by binding to transforming growth factor (TGF-α) and epidermal growth factor (EGF), and modulates cell proliferation, adhesion, transcription, migration, and angiogenesis [63,64,65,66,67,68]. EGFR is found to be expressed aberrantly in many solid tumors like lung [69], colorectal cancers [70], head and neck squamous cancers [71], and breast cancers, where around 25–30% cases are attributed to EGFR overexpression [72,73,74]. Excessive clusters of EGFR (100 times more than normal cells) in the extracellular domain of breast cancer cells [59] and a higher binding affinity towards EGF make it a rational target for its ligand.

Shimada et al. reported a 2-methacryloyloxyethyl phosphorylcholine (MPC) polymer functionalized with EGF for targeted delivery of paclitaxel [75]. Paclitaxel was successfully used in the treatment of various cancers like breast, lung, and head and neck cancers [76]. As an emulsifying agent, Cremophor EL (CrEL) is required to make intravenous (IV) formulation of paclitaxel owing to its high hydrophobicity. However, CrEL caused hypersensitivity and neuropathy in patients, so additional premedication and long-term infusions are required to counter this unwanted effects [77,78]. Thus, poly (MPC-co-n-butyl methacrylate-co-p-nitrophenyloxycarbonyl poly (ethylene glycol) methacrylate) (PMBN) comprising a hydrophobic head was constructed to incorporate paclitaxel and to increase its water solubility in the absence of CrEL. The cytotoxicity of EGF-modified PMBN-paclitaxel complexes (EGF-PMBN-PTX) was evaluated in the EGFR-overexpressed or -deficient BT-20 breast cancer cell line, A431 squamous carcinoma cell line, and H69 small cell lung cancer cell line. EGF-PMBN-PTX at different concentrations of PTX exerted significant inhibitory effects in EGFR overexpressing A431 and BT-20 cells except in EGFR-deficient H69 cell lines. In the animal model study, a suspension of 1.0 × 10^6^ A431 and H69 cells was subcutaneously administered into balb/c, nu/nu, athymic mice (15–20 g), and 15 mg/kg of PTX was given intraperitoneally for five consecutive doses in 5 days. In the A431 tumor mouse model, EGF-PMBN-PTX suppressed tumor growth significantly compared to PMBN-PTX and saline. On the other hand, in EGFR-deficient H69 tumor-bearing mice, there was no significant difference in antitumor effects between EGF-PMBN-PTX and PMBN-PTX formulations [75].

To improve the circulating time of EGFR-targeted NP-drug conjugates, NPs were modified with a hydrophilic polymer, polyethylene glycol (PEG), to reduce aggregation of NPs, to avoid RES recognition, to prevent immunogenicity, and to extend blood half-life in the systemic circulation [79,80]. Milane et al. reported an altered biodistribution and favorable pharmacokinetic profile of targeted poly(d,l-lactide-co-glycolide)(PLGA)-poly(ethylene glycol)(PEG) and poly(ε-caprolactone)(PCL)-loaded ionidamine/paclitaxel complex [81]. A peptide targeting the MDR phenotype was tagged on the surface of NPs for binding with the EGFR receptor overexpressed in MDR breast cancer cells, while the drugs were loaded into the PLGA and PCL core. Paclitaxel and lonidamine were selected for codelivery against MDR breast cancer. The biodistribution and pharmacokinetic parameters of the targeted and non-targeted PLGA-PEG-PCL NPs with conjugated ionidamine and paclitaxel were analyzed in an orthotopic tumor model. Hypoxic human breast cancer cells (2 million) were injected into female nu/nu mice for tumor inoculation, followed by treatment with paclitaxel- and lonidamine-loaded EGFR-targeted NPs, paclitaxel- and lonidamine-loaded non-targeted NPs, and paclitaxel and ionidamine solution. Quantitative and qualitative analyses of biodistribution of targeted and untargeted drug-NP conjugates demonstrated similar distributions to the liver and kidney and improved accumulation in the target tumor site without any significant off-target distribution compared to free paclitaxel and ionidamine formulations. On the other hand, pharmacokinetics parameters of targeted NPs showed augmented plasma and tumor half-life of paclitaxel and ionidamine compared to non-targeted formulations at a certain period [81].

Likewise, polymeric unimolecular micelles functionalized with a binding peptide provided an effective means of delivering the chemotherapeutic agent aminoflavone (AF) into triple negative breast cancer (TNBC). Unimolecular micelles consist of a multi-arm star amphiphilic block copolymer, poly (amidoamine)–polylactide–poly (ethylene glycol)–OCH_3_ (PANAM-PLA-PEG-OC_3_). The balance between hydrophilic and hydrophobic portions of unimolecular micelles made them stable in blood pH, with higher drug loading capabilities than conventional multilamellar micelles [82,83,84,85]. Aminoflavone, a synthetic flavonoid with significant antitumor effects [86,87], was selected and coupled to the unimolecular micelle PANAM-PLA-PEG modified with EGFR-targeted peptide GE11 (amino acid sequence YHWYGYTPQNVI). The ability of the PANAM-PLA-PEG-AF with or without targeting the peptide to mediate antiproliferative effects was evaluated in EGFR-overexpressing human TNBC cells, MDA-MB-468, and EGFR-deficient BT 474 cells, showing significant cytotoxic effects by targeted PANAM-PLA-PEG-AF in EGFR overexpressing cells compared to EGFR-negative cells. In an animal model study, 1 × 10^6^ of MDA-MB-431 cells were injected into athymic nude-Foxn1nu 5–6-week-old mice. After developing a palpable tumor, the mice were treated with saline as a control, free AF, and AF-loaded micelles targeted or non-targeted at a dose of 7 mg/kg of body weight.

The aminoflavone-loaded PANAM-PLA-PEG-AF-GE11 showed enhanced accumulation and extended plasma half-life in comparison with free aminoflavone and AF-micelles and higher inhibition of tumor growth in a mouse model of breast cancer compared with AF-micelles, without detecting any toxicity in mice [72].

Apart from the above studies, Jin et al. reported biocompatible PEG-grafted polymeric NPs conjugated with EGFR-targeting GE11 peptides for the intravenous delivery of curcumin to effectively kill breast cancer cells [88]. Curcumin (Cur) was found effective against a wide variety of diseases like neurogenerative disorder and colorectal cancer and was investigated in phase 1 clinical trial [89,90,91]. The small size range of CUR-PLGA-PEG NPs (210 nm) prepared by emulsification and the evaporation method, and the targeting efficacy of GE11 peptides allowed for maximum accumulation of curcumin in breast cancer cells. After confirming EGFR expression in MCF-7 cells by immunofluorescence study, the MCF-7 cells were treated with targeted NP-Cur and free Cur. GE11-conjugated, Cur-loaded NPs showed remarkable cytotoxic effects (<50% of control). Furthermore, the antitumor efficacy of Cur-NPs with or without targeting moiety was evaluated in an orthotopic breast tumor model created by injecting 10^7^ MCF-7 cells in female balb/c 10–12-week-old mice. PBS as a control, free Cur, Cur-NPs, and GE11-Cur-NPs were given intravenously at a dose of 5 mg/kg of Cur, and the tumor volumes were regularly measured. There was a drastic reduction in tumor growth observed with GE11-Cur-NPs. Tumors in the GE11-Cur-NPs were found to be significantly smaller, with a 50% reduction in tumor volume and weight relative to the control group [88].

### 3.2. Folate Receptor-Targeted Drug-Loaded NPs

Folic acid is an indispensable precursor for the synthesis of purine and pyrimidine and metabolism of amino acid, thereby regulating cellular growth, proliferation, and survival [58,92]. Cancer cells require more folic acid for their proliferation than the normal cells. As folic acid is not produced endogenously, cancer cells utilize folate receptor (FR) for folic acid uptake for their development and maintenance. To date, four types of folate receptor isoforms have been reported including FRα, FRβ, FRγ, and FRδ, among which FRα and FRβ are found in the plasma membrane and possess higher affinity towards folic acid (binding affinity 0.34 nM) [93,94,95]. In a solid tumor, a higher level of FR expression was found on the surface of cancer cells, and at the advanced stage, the density of FR seemed to be increased compared to normal tissues [93,96,97,98]. The location of FR, density, distribution pattern, higher folic acid absorbing capacity, and specificity towards folic acid make it a potential target for nanotherapeutics.

Folic acid (FA) was employed to design a PEG-grafted liposome-FA complex for the targeted delivery of celasterol (Cs) and irinotecan (Ir), with notable antitumor efficacy in a murine breast cancer model [99]. Targeted drug-loaded liposomal formulation was synthesized by the thin-film hydration method using dipalmitoylphosphatidylcholine (DPPC), cholesterol, and 1,2-distearoyl-sn-glycero-3-phosphoethanolamine-N-(amino(polyethylene glycol)-2000) (DSPE-PEG-NH2) and was complexed with celastrol (Cs) and irinotecan (Ir). Cs and Ir with loading efficiencies of 28.5% and 14.7%, respectively, are supposed to be assembled within the lipid bilayer part (CS) and hydrophilic core (Ir). The inhibitory effects of Lipo/Cs/Ir-FA on cell proliferation were tested in FA-positive or -negative cells, MCF-7, MDA-MB-231, and A549, with Lipo/Cs/Ir-FA showing the highest cytotoxicity (more than 80%) compared to free drugs and non-functionalized lipo-Cs-Ir formulations in FR-positive cells, MCF-7 and MDA-MB-231, whereas there was no significant difference in the cytotoxicity of both targeted and non-targeted formulations observed in FR-deficient cells, A549. The breast tumor model was created by subcutaneous injection of 1 × 10^7^ MDA-MB-231 cells into female balb/c mice and treated with control, free Cs or Ir, Cs/Ir, lipo-Cs/Ir, and Lipo-cs/Ir-FA four times at an interval of three days. A protective effect against the tumor growth was noticed in the mice treated with lipo-Cs/Ir-FA, reducing approximately 85% of the tumor volume in comparison to the control and 50% more than the non-functionalized formulation, without any significant systemic toxicity. Besides, in vivo image-guided biodistribution study revealed considerable tumor accumulation of targeted Lipo-PEG formulation compared to ligand-free formulation after 24 h of injection [99].

Although liposome proved its efficacy as an effective drug delivery vehicle, its insufficient payload capability, premature drug release, and instability raise concerns for safer human exposure [100,101,102]. To date, a combination of liposome and polymeric NPs has been developed to improve the stability and drug-loading capability, pharmacokinetics profile, and antitumor efficacy in breast cancer [103]. FA-modified liposome and polymeric NP complexes (FA-LP-NPs) comprising a lipid monolayer shell on the surface and polymeric core as a nanoscale skeleton was prepared by the thin-film hydration method and ultrasonic dispersion method using poly(ε-caprolactone)-poly (ethylene glycol)-poly(ε-caprolactone) (PCL-PEG-PCL) and 1,2-distearoyl-sn-glycero-3-phosphoethanolamine-N-(methoxy (polyethylene glycol)-2000) (DSPE-PEG 2000) and was shown to provide better therapeutic performance in terms of delivering drug at the target site. Folate receptor-expressing mammary carcinoma cells, EMT6, and FR-deficient fibroblast cells, L929, were chosen to evaluate the antiproliferative effects of the FA-LP-NP-paclitaxel (PTX) complex at concentrations of 0.25, 2.5, 12.5, and 25 µg/mL of the drug (PTX) using MTT assay after 24, 48, and 72 h of treatment. PTX-loaded FA-LP-NPs caused a significant decrease in the viability of EMT6 cells compared to LP-NP-PTX formulation without a targeting moiety at a higher exposure period. To verify the enhanced anticancer effect of PTX-loaded FA-LP NPs in the animal model, PTX-loaded LP-NPs and PTX-loaded FA-LP-NPs were given intratumorally at doses of 20 mL/kg of PTX in balb/c female mice (18–25 g) bearing tumors (inoculated by injecting 2 × 10^7^ EMT6 cells). The tumor growth trend of different treatment groups was much slower than the control group. There was approximately 20% more increase in tumor growth inhibition for targeted PTX-LPNPs (tumor growth inhibition rate of 65.78%) than FA-free formulations (tumor growth inhibition rate of 48.38%) without any toxicity [103].

Nguyen et al. reported smart NPs, combining photothermal therapy and chemotherapy for effective and synergistic antitumor efficacy in a breast cancer tumor model [104]. NIR (near-infrared) irradiation with chemotherapy is considered more efficacious against breast cancer cells as it is expected to increase local heating and mediate NIR-responsive drug release, thereby enhancing antitumor efficacy of chemotherapy. Doxorubicin (DOX) and nanosized gold nanorods (AuNR) were incorporated into the aqueous layer of the liposome (LPs), followed by functionalized with FA moiety. In vitro cellular uptake and cytotoxicity assay of FA-AuNRs-LPs were investigated in 4T1 cells with or without NIR irradiation, showing an enhanced cellular uptake and cytotoxicity in folate receptor-overexpressed cancer cells compared to the non-targeted NPs, while the in vivo study revealed a more vigorous antitumor activity than the control NPs. NIR-irradiated FA-AuNRs-DOX-LPs exerted higher cytotoxic effects (IC_50_ value of 1.90 ± 0.12 µg/mL) than the non-targeted formulation. Tumor-bearing mice were treated with PBS, free DOX, AuNRS-DOX-LPs, FA-AuNRs-DOX-LPs, FA-AuNRs-LPs + NIR, and FA-AuNRs-DOX-LPs with NIR at a dose of 2.5 mg/kg of DOX. Exposure of an NIR (1.5 w) laser for 5 min after 48 h of treatment was done for NIR treatment groups. FA-AuNRs-DOX-LPs with NIR exhibited significant tumor regression with approximately 80% reduction in tumor volume, whereas the same targeted formulation without any NIR showed approximately 64% reduction compared to AuNRs-DOX-LPs, indicating that NIR irradiation might release more DOX, which eventually increased the therapeutic responses of the drug. Bodyweight and H&E immunostaining of major organs did not show any sign of toxicity during the experimental period [104].

Polymeric NPs have emerged as a potential vehicle for the delivery of chemotherapeutic drugs, with a few currently being under investigation in clinical trials for the management of several cancers [60,105]. Tavassolian et al. delivered Docetaxel (DTX) to breast cancer cells by poly (l-g-glutamyl glutamine) (PGG) conjugated with FA [106]. FA-DTX-PGG displayed 50% more cellular internalization than the non-targeted formulations and more excellent cytotoxic effects (IC_50_ value of 0652 ± 0.027 in MCF-7 and 0.08 ± 0.02 in 4T1 cells at 72 h) than the conventional Taxotere formulation in FR-overexpressed MCF-7 and 4T1 cell lines, whereas in FR-deficient A549 cells, the targeted formulation did not show any notable uptake and anticancer effects. For evaluating the antitumor action and safety profile of PGG-FA NPs with loaded DTX, a 4T1 cells-induced murine breast tumor model was created by injecting 1 × 10^5^ 4T1 cells into female balb/c 6–8-week-old mice of. When the tumors appeared to be palpable (100–200 mm^3^), the mice were treated with Taxotere (10 mg/kg) (control), PGG-FA-NP-DTX (10 mg/kg), and PBS (control). Targeted PGG-DTX-NP formulation suppressed significant tumor growth, reducing 62% of the tumor volume compared to the conventional Taxotere formulation and 90% of the tumor volume compared to the PBS treatment, without any significant changes in body weight. Biodistribution studies revealed minimal off-target distribution to major organs and increased the accumulation of DTX in the tumor region at different time points, suggesting heightened antitumor effects and reduced unintended distribution of DTX compared to the conventional formulation [106].

In another study, Alibolandi and coworkers utilized a biocompatible theranostic nanosystem functionalized with folate moiety for the delivery of DOX to breast adenocarcinoma cells [107]. Amphiphilic copolymer was used to formulate theranostic NPs owing to its capability to load multiple imaging agents along with (both hydrophilic and hydrophobic) drug cargos [108]. FA-conjugated PEG and poly (d, l-lactic-coglycolic acid) (PLGA)-based polymersome mixed with mercaptosuccinic acid (*MSA*)-capped CdTe quantum dots (QDs) were employed to prepare FA-QDs complexes which were loaded with DOX by using a double emulsion method. Targeted FA-QD-DOX-NPs showed significantly higher cytotoxic effects in folate-overexpressed MCF-7 (IC_50_ value of 1.2 µg/mL) and 4T1 (IC_50_ value of 2.5 µg/mL) than other tested formulations, with enhanced cellular internalization, while QDs alone did not show any cytotoxic effects on breast cancer cell lines, indicating their biocompatibility and safety for carrying the drugs into the human body. In order to verify in an animal study the strong anticancer effects of FA-QDs-DOX-NPs observed at the cellular level, biodistribution and tumor regression study was performed in a mouse breast cancer model (developed by injecting 2 × 10^5^ 4T1 cells in female balb/c mice of 18–20 g). Tumor-bearing mice were given saline, free QDs, free DOX, FA-QD-DOX-NPs, and QD-DOX-NPs at a dose of 7 mg/kg DOX and 18 mg/kg of QDs. The superior antitumor effect of FA-QD-DOX-NPs might be attributed to the sufficient uptake of the nano-formulation by tumors following binding with the folate receptor and the enhanced effect of DOX on inhibition of the tumor. Toxicity markers, body weight measurement, H&E histopathology study, cardiac toxicity, and survival study showed apparently no systemic toxicity [107].

The same research group (Alibolandi et al.) further modified FA-PEG-PLGA polymeric vesicles by replacing PEG with dextran (Dex) [109]. Although PEG is widely used to increase the blood circulation time of PEGylated formulation, repeated administration of PEG may cause fast blood clearance [110]. In this regard, Dex, a natural hydrophilic polysaccharide with higher NP stabilization capabilities than PEG [109], has been applied as an alternative to PEG. FR-overexpressed MCF-7 and 4T1 cells were chosen to evaluate the antiproliferative effects of targeted or untargeted Dex-PLGA-DTX formulations and commercially available Taxotere formulation. Excellent cytotoxicity was observed for targeted FA-Dex-PLGA-DTX NPs in both MCF-7 cell lines (IC_50_ value of 4.5 µg/mL) and 4T1 cell lines (IC_50_ value of 8.3 µg/mL) than NP-DTX without FA and Taxotere formulations. The tumor growth inhibition capabilities of DTX-NP formulations in the presence or absence of FA were assessed in the 4T1 metastatic mouse breast cancer model (created by injecting 5 × 10^5^ 4T1 cells in female balb/c mice at 17–20 g). After getting palpable tumors (20–30 mm^3^), mice were treated with Taxotere (10 mg/kg), targeted or non-targeted DTX-loaded polymersomes (10 mg of DTX/kg), and saline as the control. FA-DTX-NPs significantly delayed tumor growth rates which were 83% and 90% smaller than DTX-NPs and commercially available Taxotere formulations, respectively, without any systemic toxicity. A pharmacokinetic study revealed the extended plasma concentration and greater accumulation of DTX in the tumor region of FA-conjugated NP-drug complexes than the NP-drug formulations, affirming the efficiency of FA-targeted, Dex-modified NPs for drug delivery to the specific cancer cell type [109].

### 3.3. HER-2 Targeted Drug-Loaded NPs

Human epidermal growth factor receptor-2 (HER2) is expressed excessively (100–1000 times higher than normal breast cells) to maintain the abnormal growth of breast cancer cells. Studies showed that 25–30% cases of breast cancer have HER2 gene amplification, which is responsible for the MDR effect and tumor recurrence [111,112,113]. Thus, HER2 is envisioned as a promising target for specific drug delivery to HER2-positive breast cancer cells.

In a recent study, Kumar Singh et al. explored the effectiveness of a HER2 receptor-targeting peptide by conjugating it with chitosan-modified liposome with loaded capecitabine (CAP) [114]. The surface of the CAP-loaded liposome was modified with chitosan and tumor homing peptide (THP) for selective targeting of the HER2 receptor of breast cancer cells to minimize the off-target effects of conventional CAP. The cellular internalization efficiency of the targeted chitosan-modified, CAP-loaded liposome (CTHP-CAP-LPs) was found to be approximately 71% more than the non-targeted formulation. Besides, CTHP-CAP-LPs exerted remarkable cytotoxicity compared to the non-targeted formulation owing to the greater specificity and cellular uptake of the former in HER2-overexpressed MCF-7 cells. In a mouse breast tumor model (created by injecting 1 × 10^7^ MCF-7 cells), CTHP-CAP-LPs showed superior antitumor effects with 96% reduction of the tumor volume and extended mouse survival compared to the non-targeted CAP formulation. The dose of CAP used was 20 mg/kg of the mouse body weight [114].

In another study, the importance of composition and density of the tumor-targeting peptide for delivery of the NP-drug complex was explored in HER2-positive breast cancer [115]. The composition of targeting peptide moiety was tuned to investigate the effects in binding avidity as some researchers earlier reported notable effects of composition and density of targeting peptides on receptor affinity and cellular uptake [116,117,118]. A cyclic peptide (HER2PEP) to target the HER2 receptor was selected and conjugated with 3 Lysin (K3) through the EG2 linker to improve hydrophilicity. Ethylene glycol was used to link the peptide and lipid moiety to prepare HER2PEP-k3-EG_18_ and HER2PEP-k3-EG_8_ with varied densities for evaluation in vitro and in vivo. In vitro cellular uptake study in HER2- overexpressed cell lines showed that HER2PEP-conjugated targeted nanoparticles (TNP^HER2PEP^) with 1% of peptide density had approximately 30-fold more uptake than TNP^HER2PEP^ with a density of 0.5%. On the other hand, TNP^HER2PEP^[EG_8_] yielded approximately 15- to 16-fold higher cellular uptake than high and long linker TNP^HER2PEP^formulation. The antitumor effect of the NP-drug formulation selected based on significant cytotoxic effects in HER2-overexpressed breast cancer cell lines was further verified in the mouse tumor model. TNP^HER2PEP^ [EG_8_] with a density of 0.25–0.5% showed higher accumulation in the tumor region without any off-target distribution in major organs. DOX-loaded TNP^HER2PEP^ [EG_8_] with a peptide density of 0.25–0.5% caused 68% reduction in the tumor volume compared to the untargeted formulation in an orthotopic MMTV(Mouse mammary tumor virus)-*neu* transplantation mouse model without changing the bodyweight of treated mice [115].

The benefits of polylactic-co-glycolic acid (PLGA) NPs conjugated with HER2-specific ligand to actively target HER2 receptor overexpressed by breast cancer cells were exploited [119]. The CD-340-functionalized PLGA-DOX complexes showed high stability, improved drug loading capacity, and efficient uptake by the HER2-expressing SKBr cells compared to the PLGA-DOX formulation. The effective endocytosis-mediated cellular internalization of targeted PLGA-DOX-Ab could increase apoptosis, thus exhibiting higher cytotoxic effects than the non-targeted formulations in SKbr-3 cells. The effects were less prominent in HER2-negative MCF-7 and MDA-MB-431 cell lines. On the other hand, a tumor regression study was carried out in a murine breast tumor model (induced by injecting 1 × 10^7^ SKBR-3 cells). The tumor-bearing mice were provided with saline control, free DOX, DOX-NPs, and DOX-Ab-NPs. The tumor growth curve analysis showed maximum antitumor effects exerted by targeted DOX-Ab-NPs with 38% reduction in the tumor volume compared to the DOX-NPs. Moreover, the extended tumor accumulation, less off-target biodistribution in major organs, and significant reduction of cardiac accumulation affirmed the specific targeting capability of DOX-Ab-NPs in the animal model study [119].

In an attempt to increase the drug ability by overcoming receptor heterogenicity, Houdaihed et al. developed a polymeric NP functionalized with Fab fragments to target HER2 and EGFR receptors for the delivery of paclitaxel (PTX) and Everolimus (EVER) in both monolayer and 3D breast cancer cells [120]. Dual targeted NP formulation was prepared by attaching trastuzumab (TmAb) Fab fragments (for targeting HER2) and panitumumab (PmAb) (for targeting EGFR) obtained from the digestion of full Ab by papain to PEG-b-PLGA NPs with loaded drug cocktail (PTX + EVER) through the amino coupling. Cellular uptake and cytotoxicity studies were performed in SkBR3 (HER_high_/EGFR_med_) and MCF-7(HER_neg_/EGFR_low_) breast cancer cell lines. In SkBR3 cells (HER_high_/EGFR_med_), the rate of internalization of dual-targeted NPs was higher than the non-targeted formulation (71.6 ± 7% vs. 57.6 ± 3.2%). On the other hand, there was no significant difference in cellular uptake pattern for both dual and single-targeted NPs in poorly expressed MCF-7 (HER _neg/_EGFR _low_) cells, indicating that dual-targeted PEG-b-PLGA NPs could be taken up by SkBR3 cells more rapidly, which was further confirmed by measuring cellular uptake after blocking either EGFR or HER2 receptors. In agreement with cellular internalization pattern, PTX-EVER-loaded dual-targeted NPs showed maximum toxicity against SKBR3 cells compared to the drug-NP complexes without any ligand, whereas there were no significant changes among the formulations tested in MCF-7 cells. Furthermore, the antitumor response of dual NP-loaded PTX-EVER conjugates was evaluated in 3D spheroid models of SkBR3 cells and MDA-MB-436 (HER_neg_/EGFR_low_) cells. The percentage of change in the size of spheroid for dual NPs was approximately 84% less than the untargeted NPs in SKBR3 cells. In contrast, in MDA-MB-456 cells, there was no significant shrinkage of spheroid for both formulations with or without targeting agents [120].

### 3.4. Estrogen Receptors-Targeted Drug-Loaded NPs

Estrogen receptors are a member of the nuclear receptor superfamily involved in growth and development of many organs including the bone, brain, and cardiovascular and female reproductive systems, e.g., ovary, breast, and endometrium [121,122,123]. Estrogen hormone acts by binding with estrogen receptors, ERα and Erβ, located on tissue or cells and plays a vital role in cancer development. In healthy breast cells, ERα expression is less than 10%, but significant upregulation of ER expression (80%) is found in breast cancer cells [124,125,126,127]. The elevated expression of estrogen receptors in breast cancer makes them potential targets for specific delivery of cytotoxic agents.

Tang et al. developed epirubicin (EPI) and PTX-encapsulated, estrogen-functionalized pegylated liposomes to enhance the antitumor efficacy of the drugs against breast cancer cells while reducing their adverse off-target effects [128]. The monodisperse liposomes with average sizes of around 120 nm and PDI of more than 0.02 were prepared by adding soy phospholipids (SPC), cholesterol (CHO), and DSPE.mPEG_2000_ and were functionalized with estrone (ES). This targeted nanoformulation having an excellent drug loading capability showed a significant cellular uptake and release profile. Based on an in vitro cytotoxicity study on MCF-7 cells, ER-targeted SSL-EPI/PTX was injected into tumor-bearing mice and found remarkable tumor growth inhibitory effects on breast cancer without any visible systemic toxicity. The targeting capability of ES and internalization of a high concentration of EPI and PTX could enhance the therapeutic responses of EPI and PTX against breast cancer cells [128].

Recently, Mamnoon et al. developed hypoxia-responsive polymeric NPs (HRPs) functionalized with 17β-estradiol (E2) for the targeted delivery of DOX in ER-positive breast cancer cells [129]. Estradiol conjugated polymersome-DOX complex (E2-DOX-HRPs) was prepared by the solvent exchange method with a diameter of 168 ± 3 nm and a high loading efficiency for DOX (59%) compared to the non-targeted (48%) formulation. In hypoxic condition, the diazobenzene of polymer was reduced through the reductase enzyme present in the tumor microenvironment and destabilized the polymeric structure, thereby triggering release of the drug. The E2-conjugated DOX-HRPs were significantly taken up by MCF-7 cells and showed augmented antiproliferative effects in hypoxic conditions compared to the E2 free HRP formulation (86% cytotoxicity of targeted HRPs vs. 61% cytotoxicity of non-targeted HRPs). In a three-dimensional spheroids culture of MCF-7 cells, hypoxia-responsive E2-DOX-HRPs resulted in notable reduction of cell viability (21%) and shrinkage of spheroid compared to the DOX-HRP formulation in hypoxic condition. On the other hand, in the normal condition, there was also a reduction of cell viability in the group receiving ER-targeted HRP formulation (55%) but no significant changes were seen in the group receiving ER-free DOX-HRP formulations. These results indicated that the targeted HRP formulation successfully recognized the estrogen receptor of breast cancer cells and released a higher amount of DOX in the hypoxic conditions of the tumor, thereby augmenting the anticancer effects of DOX [129].

### 3.5. CD-44 Targeted Drug-Loaded NPs

CD-44 is a transmembrane glycoprotein located on the surface of adult and fetal tissues, e.g., central nervous system, lung, and epidermis. It is the main receptor for the binding of extracellular matrix glycosaminoglycan hyaluronan (hyaluronic acid), osteopontin (OPN), collagen, and matrix metalloproteinase (mmp) [130,131]. In normal healthy tissue, CD-44 regulates hyaluronic metabolism, lymphocyte activation, and cytokine release. A higher expression of CD-44 is found in many cancer types and the elevated level of CD-44 in serum is used as a diagnostic marker for estimation of the tumor burden in colon and gastric cancers [132]. In breast cancer, CD-44 is expressed aberrantly and the level of CD-44 expression increases proportionally to the grade and stages of invasive breast tumor [131]. Therefore, CD-44 can be a suitable target for the delivery of anticancer agents specifically to breast cancer cells.

CD-44 receptor-targeted, DOX-embedded hybrid micelles intensified the antitumor efficacy in the 4T1 mouse model, shedding light on the potential application of the combined strategy for management of triple negative breast cancer [133]. The hybrid HL-LT was prepared by combining hyaluronic acid (HA)-D-α-tocopheryl succinate (HA-TOS, HT) with low molecular weight heparin-TOS (LMWH-TOS, LT) micelles with ester bond, and coupled with DOX embedded in the hydrophobic core of the micelles and HA or LMTH l localized on the shell to avoid opsonization in the systemic circulation. Targeted hybrid HL-LT with loaded DOX showed 2.2 times higher uptake in CD-44-expressed cell line 4T1 compared to normal N111373 cells, suggesting receptor-mediated cellular internalization. The cell proliferation inhibition efficacy of targeted DOX-NPs exhibited a strong dose-dependent pattern, with the maximum inhibition of tumor cell proliferation found at a higher concentration of DOX in CD-44-expressed cell lines. The enhanced anticancer effects of targeted DOX-NPs were further verified in the breast cancer mouse model. An in vivo biodistribution study showed significant accumulation of DOX in the tumor region of targeted HT-LT-DOX-NPs compared to other tested formulations, while the treatment with targeted HT-LT-DOX-NPs led to more effective inhibition of tumor growth (85%) than free DOX treatment in tumor-bearing mice. Moreover, the bioluminescence imaging study revealed that the targeted nano-formulation stopped metastasis of tumor cells in the lungs very significantly. Histopathology study and regular body weight measurement of tumor-bearing mice did not show any notable changes, suggesting the absence of any serious adverse effects of targeted NPs on major organs [133].

In another study, Li et al. attempted to produce the NP-drug complex by a one-step method to circumvent conventional multistep synthetic procedures for making it more feasible for large-scale production [134]. CD-44 receptor-targeted HA was functionalized with Carbon Dots (CDs) loaded with DOX by using an acid sensitive linker, 4-carboxybenzaladehyde (P-CBA), to accelerate the release of DOX from the vehicle in an acidic environment. Viability of CD-44-expressed 4T1 cells decreased significantly by targeted HA-CD-P-CBA-DOX complexes compared to the blank. The anticancer efficacy of HA-CD-P-CBA-DOX was tested in a balb/c mouse 4T1 tumor model established by injecting 1 × 10^7^ 4T1 cells. After getting a palpable tumor (50–100 mm^3^), tumor-bearing mice were treated with PBS and free DOX at a dose of 3 mg/kg. The group of mice receiving HA-CD-P-CBA-DOX delayed tumor growth, which had 77% and 88% reductions in tumor volume compared to CD-P-CBA-DOX and the control. The superior antitumor effect of HA-CD-P-CBA-DOX might be ascribed to HA-mediated sufficient cellular uptake in the tumors; after being internalized, DOX was released quickly in the acidic environment of endosomes and resulted in an enhanced effect of DOX on tumor inhibition. Blood biochemical index, bodyweight, and H&E study of the major organ of mice showed no significant changes and toxicity of the treated mice [134].

To improve the targetability of NP-loaded drug complexes, Seng et al. reported dual-targeted redox-responsive hybrid polymeric micelles functionalized with HA to improve the delivery efficiency of an NP-loaded anticancer drug, Gambogic acid (GA)[135]. A self-assembled nanosystem composed of an outer layer of PEGylated HA for targeting the CD-44 receptor, reduction-responsive disulfide-linked hexadecanol (HEX), chitosan (CHO), and superparamagnetic iron oxide nanoparticles (SPION) as a core to load GA. The passive targeting capability of SPION and receptor-mediated endocytosis allowed for higher cellular accumulation and enhanced internalization of the mPEG-HA/CHO-SS-HEX-SPION complex in overexpressed 4T1 cells. The half maximal inhibitory concentration of mPEG-HA/CHO-SS-HEX-SPION-GA was found to be lower than that for other formulations which were 87%, 85%, and 83% lower than free GA in the MCF-7, MDA-MB-231, and 4T1 cell lines, respectively, suggesting significant antitumor efficacy of targeted NP-drug complex at the cellular level. An in vivo mouse model was created by injecting 4T1 cells and treated with saline as a control, free GA, CHO-SS-HEX-SPION-GA, mPEG-HA/CHO-SS-HEX-SPION-GA-MF, and mPEG-HA/CHO-SS-HEX-SPION-GA + MF. The dose of GA was 6 mg/kg, and a magnetic field (MF) of strength 0.2 T was applied to the tumor position. The tumor volume treated with mPEG-HA/CHO-SS-HEX-SPION-GA + MF was significantly smaller than the tumor volume treated with free GA. There was a 88% reduction in the tumor volume for mPEG-HA/CHO-SS-HEX-SPION-GA with MF compared to free GA formulations. A biodistribution and toxicity study with the nano-formulation revealed extended concentrations of the drug in plasma without any notable toxicity [135].

### 3.6. Transferrin-Receptor Targeted Drug-Loaded NPs

Transferrin (Tf) is a β-globulin that facilitates iron transport in the body through transferrin receptors. Ferric ion (Fe^3+^) is transported into cells via transferrin receptor-mediated endocytic pathway. After endocytosis, Fe^3+^ dissociates from the transferrin complex at late endosomal acidic pH (<6.00) [136,137,138]. The Tf receptor (TfR) is highly expressed (2–10 times more than normal cells) in almost all cancers like breast cancer, lung cancer, and lymphoma [138,139]. Consequently, a higher binding affinity of Tf to TfR, a known internalization and recycling mechanism of TfR, the correlation between overproduction of TfR, and development of MDR present TfR as a promising drug target for delivery of anticancer agents to the breast cancer cells.

Cui et al. demonstrated that a combination of anticancer drugs, DOX, and curcumin (CU) loaded into targeted NPs exerted superior anticancer effects over monotherapy without any systemic toxicity [140]. The targeted dual drug-NP complex was prepared by mixing PEG-aldehyde (CHO) and adipic acid dihydrazide (ADH) attached to CU by a pH-sensitive linker, hydrozon bond, and DOX was loaded in the core of the self-assembled NPs. Targetability and anticancer effects of the stimuli-responsive prodrug-based nano-assemblies were tested in transferrin receptor-overexpressed MCF-7 cells and compared with free DOX and conventional liposomal DOX formulations. Tf-modified PEG-CUR/DOX-NPs had lower IC value (2.5 µm) than the other tested formulations. To evaluate the synergistic effects of DOX and CUR in animal studies, Tf-PEG-CUR/DOX-NPs were injected in female balb/c mice with tumors inoculated by injecting 1 × 10^6^ MCF-7 cells. The dose used for the antitumor evaluation was 50 mg/kg of CUR and DOX once a week with a total of seven doses. Compared to liposome-DOX and free DOX, Tf-PEG-CUR/DOX-NP complexes strongly inhibited tumor growth. The inhibition rates of tumor growth (IRT) were found to be 83.5%, 53.2%, and 37.9% in the mice receiving Tf-PEG-CUR/DOX-NPs, liposome-DOX, and CUR-DOX formulations, respectively. The PEGylated Tf-CUR/DOX-NPs had longer blood circulation time and higher drug accumulation in the tumor region without any significant toxicity, confirmed by biodistribution and toxicity studies [140].

In another study, TfR-targeted pH-responsive hybrid (HD) micelles were designed for the delivery of DOX to counter the MDR of breast cancer cells [141]. A TfR ligand, HAIYPRH (7 pep), was chosen to target TfR owing to its low immunogenicity. The mixed micelles were synthesized by mixing of poly(l-histidine)-coupled polyethylene glycol-2000 (PHIS-PEG_200_) and 1,2-distearoyl-sn-glycero-3-phosphoethanolamine-polyethylene glycol-2000 (DSPE-PEG_200_) and were modified with 7 pep to form 7 pep-DSPE-PEG_200_ (7 pep HD micelles). The drug efflux test and internalization assay of 7 pep-DSPE-PEG_200_ showed lower intracellular level and the highest cellular uptake compared to the non-targeted and free DOX groups, suggesting override of the MDR-mediated drug efflux and TfR-mediated enhanced cellular uptake in TfR-overexpressing MCF-7 cells. Free DOX at higher concentrations showed very low cytotoxicity due to MDR of MCF-7/adr tumors. On the other hand, 7 pep HD micelles-DOX complexes significantly inhibited the growth of tumor cells even at lower drug concentrations (0.1 and 0.5 µg/mL). Furthermore, the tumor-targeting ability of 7 pep HD micelles with a loaded near-infrared fluorescent probe, DiR, in female balb/c mice bearing MCF/adr tumors was investigated using in vivo imaging technique. The 7 pep HD micelles 24 h after injection showed significant and enhanced tumor accumulation, which was approximately 2 times higher than the non-targeted formulation [141].

### 3.7. αvβ3 Integrin-Targeted Drug-Loaded NPs

As a member of the cell adhesion molecules involved in cell–cell and cell–ECM interactions, αvβ3 integrin plays a vital role in cell morphology, locomotion, mitosis, cytokinesis, migration, and phagocytosis [142,143,144]. Usually, αvβ3 integrin is not present in pre-existing endothelial cells and normal tissues but is found to be overexpressed on the surface of breast tumor cells [145,146]. In tumor cells, αvβ3 plays a role in destroying the basement membrane and interstitial matrix through activation of MMP-2 and plasmin, which may accelerate tumor invasion and metastasis [147]. αvβ3 integrin that possesses a high affinity towards RGD-containing components of ECM protein can be considered an ideal target for its ligands in order to facilitate specific delivery of anticancer agents to breast cancer cells.

Yadav et al. developed pH-responsive chitosan NPs for the delivery of Raloxifene (Rlx) to breast cancer cells and demonstrated the impact of pH on stability, cellular internalization, and anticancer effects of NP-drug conjugates [148]. A well-known RGD peptide was used to prepare targeted chitosan NPs (cRGD-CHNPs) incorporating the anticancer drug Rlx. RGD-CHNPs of 200 nm showed high stability at acidic pH, high encapsulation efficiency (50%) for Rlx, and improved cellular internalization in 4T1 and MDA-MB-231 breast cancer cells. The targeted Rlx-RGD-CHNPs showed higher cytotoxic responses and apoptotic activity at pH 6.5 compared to physiological pH in αvβ3-expressing 4T1 and MDA-MB-231 cells. Moreover, in vivo studies in mice with 4T1 breast carcinoma revealed the enhanced antitumor efficacy of Rlx, decreasing tumor volume, which was approximately 62.5% of the tumor volume compared to untargeted formulations. No significant toxicity was observed in the hematotoxicity study, liver function test, kidney function test, and histopathology assessment, suggesting that RGD-CHNPs could be considered as a useful and biocompatible targeted drug carrier [148].

In another approach increasing the therapeutic responses of DOX against metastatic cancer cells, Covarrubias et al. developed a formulation, DSPE-PEG-NPs, with a targeting peptide for selective delivery of the drug [149]. The dual-ligand NPs prepared by conjugating αvβ3 integrin-targeting peptide c (RGDfc) and EGFR-targeting peptide (YYHWYGYTPQNVI) with PEG-NH_2_ terminals of liposomal DSPE NPs using a sufo-SMCC cross-linker showed high cytotoxicity (IC_50_ value at 0.3 µM) when loaded with DOX against metastatic D2-A1 breast cancer cells expressing EGFR and αvβ3 integrin receptors. In an animal model study, the targeting and antitumor efficacy of dual-targeted NPs was quantified by measuring the BLI signal of a central metastatic organ, the lung. Free DOX and NPs without targeting peptide at a drug dose of 7.5 mg/kg did not show any therapeutic responses against metastatic breast cancer cells. On the other hand, the metastatic outgrowth in animals treated with the dual-targeting DOX-loaded NPs was remarkably slower than the groups treated with non-targeted NPs and mono-ligand-targeting NPs. Also, the extended survival rate, unchanged body weight, and improved deposition in lungs (as confirmed by histological analysis) was found in the animal treated with targeted formulations [149].

### 3.8. Biotin Receptor-Targeted Drug-Loaded NPs

Biotin, a member of the vitamin B complex, assists in growth and development of cells. Cancer cells extensively utilize additional biotin through an overexpressed biotin-specific receptor for their rapid proliferation compared to normal tissues [150]. Thus, the biotin receptor could be an ideal target for the targeted delivery of NP-drug conjugates.

Taheri et al. described human serum albumin (HSA) nanoparticles (HSNs) coupled with Methotrexate (MTX) and functionalized with biotin molecules for the specific delivery of MTX to 4T1 breast carcinoma cells [150]. The biotinylated NP-MTX conjugates showed dose-dependent responses against tumor growth in the mouse animal model. At a high dose of MTX (12.50 mg/kg), MTX-HSA-NPs containing more biotin showed remarkable inhibition of tumor growth (approximately ten-fold lower in tumor volume than the non-targeted formulation) with an extended survival rate, suggesting that more biotin molecules allowed for more specific recognition of biotin-specific receptors on breast cancer cells, resulting in more cellular internalization of MTX and improved therapeutic responses [150].

Patra et al. developed porous hexagonal ZnO nanodisc (PZHD) for the targeted delivery of DOX [151]. The PZHD was functionalized with NHS biotin to make actively targeted biotinalyzed PZHD (PZHD-BT) while the porous structure allowed to load the maximum amount of DOX (63%). With NP-conjugated DOX, the viability of MCF-7 cells after 24 h of treatment at the highest concentration of DOX (20 µg mL^−1^) was decreased significantly to 11% compared to free DOX (25%). PZHD-BT with loaded DOX was investigated for its antitumor activity in a malignant breast tumor model created by injecting EAC cells in Swiss albino mice. When the mice had a tumor volume of 150 mm^3^, they were treated with PZHD-BT-DOX and free DOX. The tumor volume and weight of mice treated with PZHD-BT-DOX were significantly smaller than the free DOX-treated group. The cellular recognition of biotin presents on the surface of NPs through its cognate (biotin-specific) receptor enabled targeted delivery of DOX to the tumor cells, leading to the maximum tumor growth inhibitory effect compared to the untargeted formulation. Moreover, PZHD-BT showed excellent biocompatibility without showing any change in toxicological parameters and immunological activities [151].

The MDR of breast cancer cells against multiple chemotherapeutic agents is one of the biggest challenges for successful chemotherapy in the clinic. In particular, P-glycoprotein (P-gp), an ATP-binding cassette (ABC) transporter flashes out the internalized drug, thereby reducing the drug concentration in the cytoplasm of the tumor cell [152,153]. In order to address this issue, Lv et al. designed biotin-decorated poly(ethylene glycol)-b-ploy (ε-caprolactone) NPs to encapsulate a drug cocktail containing DOX and a chemosensitizer, quercetin (BNDQ) to reverse the MDR [154]. Drug-sensitive MCF-7 and P-gp-overexpressing MCF-7/ADR cells were chosen to evaluate cytotoxicity and drug efflux. The codelivery of DOX and BNDQ with or without biotinylated NPs showed significantly more anticancer effects than free DOX in P-gp-overexpressing MCF-7/ADR cells. Moreover, in a MCF-7/ADR cell-induced tumor model, accumulation of DOX at the target tumor site was found to be higher in mice treated with the targeted formulation of DOX + BNDQ than the mice treated with DOX (1.6-fold higher than non-targeted formulation), resulting in enhanced inhibition of the tumor growth (75% decrease of the tumor volume) without any systemic cytotoxicity. These results suggest that, by inhibiting both the activity and expression of P-gp in MCF-7/ADR cells, BNDQ could increase the cytoplasmic concentration of DOX, eventually improving its therapeutic responses [154]. Targeted NPs could enhance the intracellular delivery of both DOX and BNDQ to the tumor, thus enabling BNDQ to significantly inhibit P-gp and prevent DOX efflux.

### 3.9. LHRH Receptor-Targeted Drug-Loaded NPs

Produced in the hypothalamus, luteinizing hormone releasing hormone (LHRH) or gonadotropin-releasing hormone (GnRH) triggers the release of steroid sex hormones, estrogen, progesterone, and testosterone [155]. These steroid hormones promote the growth of breast cancer cells. The LHRH receptors are found to be overexpressed in many tumors including breast tumor (approximately 50%), ovarian and endometrial tumor (approximately 80%), and prostate tumor (approximately 90%) [156,157]. The aberrant expression of LHRH receptor on the particular cancer cells compared to healthy tissues renders it specific as a target molecule for tumor-targeted delivery of chemotherapeutic agents.

Li et al. exploited the LHRH peptide as a targeting moiety and examined the targeting efficiency and biodistribution of LHRH-modified cisplatin (CDDP)-loaded micellar NPs in an animal model in breast cancer [158]. The nanomicelles were synthesized by complexing dextran-modified succinic acid (Dex-SA) with CDDP and by altering the micellar surface with LHRH peptide to make targeted (Dex-SA-CDDP-LHRH) conjugates. The intracellular platinum content of targeted Dex-SA-CDDP-LHRH (8.45 ngpt/10^6^ cells) was significantly higher than non-targeted formulations (4.97 ngpt/10^6^ cells), indicating improved cellular internalization through LHRH receptor-mediated endocytosis. Dex-SA-CDDP-LHRH (IC_50_ value 106.3 µmolptL^−1^) showed higher MCF-7 cell growth inhibitory effects than Dex-SA-CDDP (IC_50_ value 146.4 µmolptL^−1^). The pharmacokinetic study was carried out in nude mice bearing MCF-7 tumors, showing extended plasma concentration of Pt and higher tumor accumulation (2-fold) than the formulation without LHRH. For tumor regression study, the tumor-bearing mice were treated with PBS, CDDP, Dex-SA-CDDP, and Dex-SA-CDDP-LHRH at drug doses of 4 mg kg^−1^ and 10 mg kg^−1^. At a higher concentration of the drug (10 mg kg^−1^), the group receiving Dex-SA-CDDP-LHRH treatment exhibited greater regression in their tumor volumes (tumor regression rates of 70%) than untreated formulation, without any systemic toxicity [158].

Later on, the same group employed Dex-SA-CDDP-LHRH for suppression of growth and metastasis of breast cancer using 4T1 aggressive metastatic breast cancer cell lines in vitro and in vivo [159]. The Dex-SA-CDDP-LHRH micelle demonstrated efficient intracellular uptake and enhanced cytotoxicity (IC_50_ value 87.95 µmolptL^−1^) compared to the untargeted formulations (IC_50_ value of 66.27 µmolptL^−1^) in LHRH-overexpressing 4T1 cells. The in vivo toxicity, tolerability, biodistribution, and tumor regression studies were performed following induction of breast tumors by injecting 1.5 × 10^6^ of 4T1 cells into female balb/c mice. Dex-SA-CDDP-LHRH showed a higher accumulation of platinum drug in primary metastatic organs, liver (1.83 fold), spleen (1.83 fold), and lung (1.47 fold) than free CDDP. The tumor-targeting ability of targeted Dex-SA-CDDP-LHRH was higher than Dex-SA-CDDP (3376.8 ± 63 ng g^−1^ vs. 2137.4 ± 334.7 ng g^−1^). Moreover, targeted formulation produced an enhanced inhibition of tumor growth at a higher dose of CDDP (10 mg kg^−1^) with a tumor inhibition rate of >70% compared to Dex-SA-CDDP (tumor inhibition rate of >60%). The metastatic capability of breast cancer cells was found to be inhibited in both the early and late stages in mice receiving Dex-SA-CDDP-LHRH [159].

Zhang et al. developed long-circulating mitoxantrone (MTO)-loaded liposomes to actively target the LHRH receptor of breast cancer cells [101,160]. These gonadorelin-functionalized DSPE-PEG_2000_ (120 nm) liposomes generated by the thin-film hydration method revealed an excellent drug loading and encapsulation efficiency (93.5%) and showed a significantly higher rate of internalization by LHRH receptor-overexpressing MCF-7 cells, resulting in increased inhibition of tumor cell growth. The concentration of MTO in blood and target tumor cells was found to be higher in MCF-7 tumor-bearing mice treated with LHRH-MTO-liposomes in contrast to non-targeted formulations. Moreover, a tumor regression study in female balb/c mice with MCF-7 breast tumor revealed that the LHRH receptor-directed, MTO-encapsulated liposomes enhanced the antitumor responses of MTO (with 38% reduced tumor volume) and reduced its toxic effects compared to the MTO-liposomes [160].

## 4. Therapeutic Evaluation and Translational Gap of Receptor-Targeted Drug Delivery

From the above studies, it is evident that receptor-targeted NP/drug conjugates have been investigated extensively to eliminate the existing adverse effects of conventional chemotherapy. Re-formulation of anticancer agents as targeted nano-chemo complexes is also highly promising to improve the therapeutic index of chemotherapeutic agents and to circumvent the MDR effects. Distinctive receptors overexpressed on the surface of the breast cancer cells could selectively bind to the targeting ligands of the drug-encapsulated nano-carriers as part of active cellular targeting strategies. These rationally designed organic, inorganic, hybrid, stimuli-responsive, and theranostic NPs were evaluated for their potential to effectively deliver anticancer drugs to tumor tissues in terms of fold-reduction in tumor size or mass compared to free drugs in animal models of breast cancer (Table 1).

**Figure 4:**
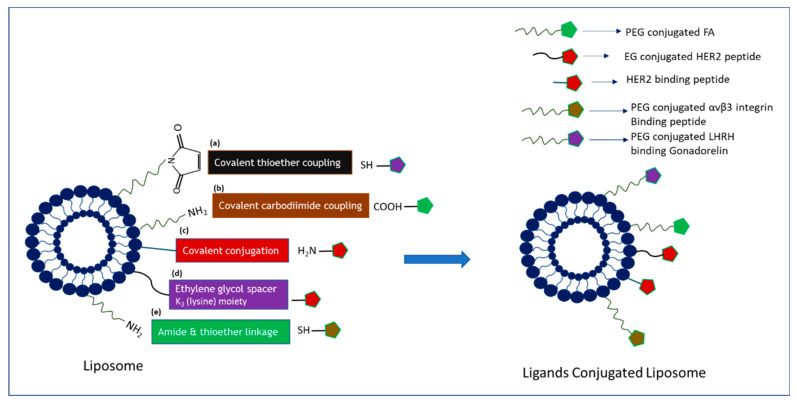
Strategies in adsorbing ligands onto the surface of liposomes: schematic representation of the reactions between the liposome bilayer containing functional groups and multiple ligands via (a) covalent thioether coupling, (b) covalent carbodiimide coupling, (c) covalent conjugation, (d) ethylene glycol spacer, and (e) amide and thioether linkage.

**Figure 5:**
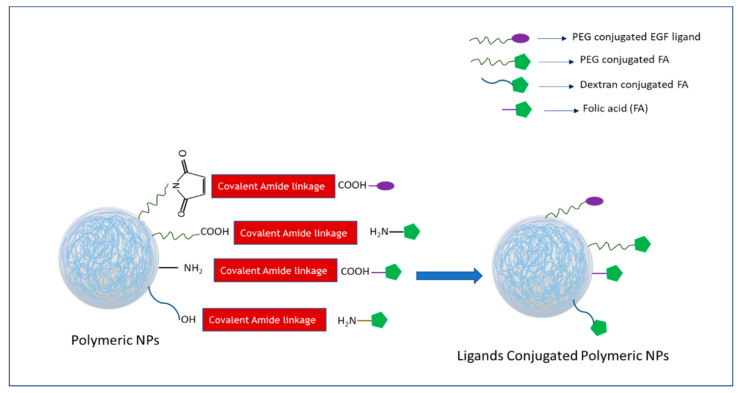
Strategies in conjugation ligands with polymeric nanoparticles: schematic representation of the reactions between the functional groups of polymeric nanoparticles and multiple ligands via covalent amide linkage.

**Figure 6:**
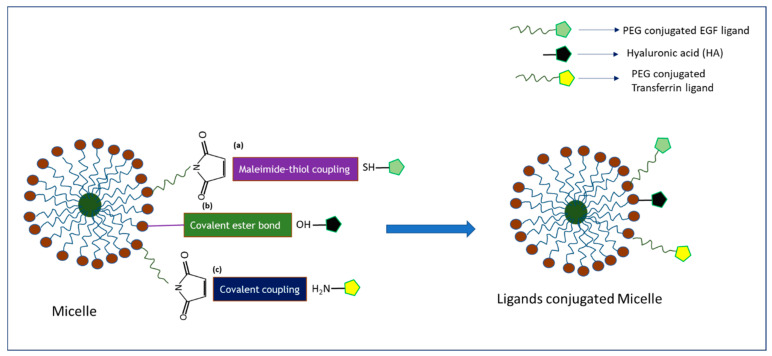
Strategies in conjugation ligands with polymeric micelles: schematic representation of the reactions between the functional groups of polymeric micelles and multiple ligands via (a) maleimide-thiol coupling, (b) covalent ester bond, and (c) covalent coupling.

Ligands-tagged nanotherapheutics synthesized using different conjugation techniques (Figure 4, Figure 5 and Figure 6) could selectively identify and bind to the receptors like EGFR, FR, Tf receptor, HER2, LHRH receptor, αvβ3, and CD-44 overexpressed on the surface of breast cancer cells and could facilitate cellular internalization through receptor-mediated endocytosis. High drug accumulation at the target tumor site corresponded to robust antiproliferative effects as a result of NP-facilitated cellular internalization of the drug in the cancer cell lines overexpressing the specific receptor. The enhanced antitumor efficacy of actively targeted nanotherapeutics found in the different breast cancer cell lines was further verified in various animal models of breast tumor. The quantitative and qualitative analyses of various targeted nano-drug complexes in animal models demonstrated altered and favorable pharmacokinetic profiles of targeted formulations compared to non-targeted ones. NP-conjugated drug complexes extended the blood half-life via exertion of stealth effects to prevent the formation of protein corona on particle surfaces and RES clearance. The particle size and surface properties of NPs might help lower drug loss, augmenting drug accumulation at the target tumor site and reducing unwanted side effects of chemotherapy. Several studies presented above showed significant deposition of drugs at the tumor site and remarkable tumor regression capacity of actively targeting nanoparticle-drug complexes compared to empty vehicles. Moreover, changes in the body weight, histology of major organs, and blood biochemical and hematological parameters of the treated mice were found negligible compared to the untreated animals.

Despite having tremendous success in preclinical studies, targeted drug delivery systems come with several hurdles which eventually limit success in clinical settings. The translational gap of targeted therapy towards the clinical use is also growing with identification of several biological and translational barriers that need to be tackled to ameliorate the clinical performances [161,162]. One of the biggest challenges is the scalability for industrial production of targeted NPs, as it is challenged by potential issues like multistep synthetic procedure; structural and physicochemical properties, i.e., stability, consistency, and reproducibility; and higher manufacturing cost [163,164]. Secondly, there is lack of adequate data on immunoreaction and toxicology studies of NPs in humans. Although these NP-drug conjugates were nontoxic in animal models, feasibility in drug dosing, dosage form, and route of administration should be assessed further before heading to the clinical trial. Finally, the selection of ideal receptors and proper ligands is also a crucial factor and remains a challenge for the rational design of actively targeting NPs. NP-drug conjugates might not be readily accessible to the target cells located far from blood vessels, for example, FR-targeted imaging agents did not enter the remotely placed FR-expressing apical membrane [62,165]. To some extent, targeting moiety may trigger cancer development, i.e., targeting moieties like vitamin B12-enhanced methylation, which increased the risk of cancer progression [166]. Besides, folic acid is not only overexpressed in cancer cells but also expressed highly in normal cells of lungs, kidney, placenta, and choroid plexus, which may cause an off-target distribution of drugs, resulting in systemic toxicity. Once the targeting NP complex accumulates in the target site, it has to compete with endogenous ligands for coupling with respective receptors, for example, exogenous transferrin competes with endogenously available transferrin to bind with the transferrin receptor. To resolve the above shortcomings, a better understanding of tumor biology and heterogeneity, molecular mechanisms of cancer development, and insights of cancer metastasis may help to select the right therapy for the right patients. Further improvement in the design and selection of NPs especially in terms of a simple and one-step production method, the stability of NPs, batch to batch reproducibility, scalability, and cost-effectiveness should be considered to improve clinical performances. Before selecting target receptors, thorough screening of receptor type and their constituents, expression and kinetics patterns in patients, and safety of the targeting moieties should be monitored closely to increase the effectiveness of targeted therapies and to mitigate off-target effects in human trials.

## 5. Conclusions and Future Prospective

In summary, receptor-targeting of nanoparticles possesses a great perspective as an ideal drug delivery approach, forming the basis of an ever-expanding area of cancer nanomedicine to circumvent the limitations of currently available chemotherapeutic agents. Vigorous research on tumor, tumor microenvironment, and metastasis resulted in discovery of many promising molecular targets for tumor targeting and therapy. Meanwhile, tremendous progress on the design of diverse nanoparticles and understanding of their multiple biological, pharmaceutical, and translational barriers introduced several smart nanocarriers and novel delivery approaches for improving the clinical impact of cancer nanomedicine. A variety of organic, inorganic, hybrid, and stimuli-responsive NPs were developed so far to deliver multiple chemotherapeutics for the management of breast cancer. For active targeting of the NPs, several ligands of different sizes and molecular entities were used to target specific receptors which are overexpressed in breast cancer cells. Preclinical animal studies demonstrated that the targeted NP-drug complexes enhanced cellular internalization and the therapeutic index of anticancer drugs and reduced unintended side effects and chemoresistance in breast cancer cells compared to free drugs. These exciting outcomes hold promise for clinical translation of therapeutic NPs. Further investigations are highly desirable in the selection of an ideal receptor and in understanding the effects of protein corona spontaneously formed on NPs in blood, the ligand–receptor interaction, the rate of internalization of drug-loaded NPs, and the drug-release mechanisms inside the target cell. Employing combinatorial therapy, dual targeting strategies, and theranostic NPs would help to improve performance of the nanotherapeutics. Finally, precisely designed animal models and the adoption of good laboratory practices (GLP) on the laboratory scale may contribute to developing clinically translatable targeted nanotherapeutics for the management of breast cancer.

## Figures and Tables

**Table 1:** Assessment of the antitumor effects of specific receptor-targeting nanoparticles carrying chemotherapy drugs following systemic delivery in animal models of breast cancer.

Constituents (1) of Nanoformulations and Their Sizes (2)	Name of Drug (1), Its Amount Used (2) and Encapsulation (3a) or Loading (3b) Efficiency	Cell Lines (1) and No. of Cells Used (2) to Induce Tumors in Animals (3) (Mice/Rats)	Days Required to Form Tumors (1) and Their Sizes (2) Prior to Administration	Route of Delivery (Intraven-Ous (i.v.)/Intratum-Oral/Peritoneal (i.p.)/Oral)	Amount of Drug Administered at One Time (1) and Frequency of Administration (2) and Interval Time (3)	Total Period of Tumor Measurement since 1st Administration	Fold Reduction in Tumor Size (1 a) or Mass (1b) Compared to Free Drug at the End	Decrease in Body Weight for Drug-Free Particles (1) and Drug-Loaded Particles (2)
(1) EGF peptide- PMBN (2) ~200 nm [75]	(1) Paclitaxel (2) 15 mg/kg and (3a) NR (3b) NR	(1) A431 and H69 (2) 1.0 × 10^6^ (3) mice	(1) NR (not reported) (2) 100 mm^3^	i.p.	(1) 15 mg/kg (2) 5 (3) 1 day	21 days	(1a) 2-fold (1b) NR	(2) No (2) No
(1) EGF peptides-polymeric micelle (2) 20–100 nm [72]	(1) Aminoflavone (2) 7 mg/kg and (3a) NR (3b) 16.7%	(1) MDA-MB-468 (2) 1.0 × 10^6^ (3) mice	(1) 56 days (2) 500 mm^3^	i.v.	(1) 7 mg/kg (2) 5 (3) 4 days	46 days	(1a) 7-fold (1b) NR	(2) NR (2) NR
(1) EGF peptides-PLGA-PEG (2) 210 nm [88]	(1) Curcumin (2) 5 mg/kg and (3a) 92.3 ± 2.7 (3b) NR	(1) MCF-7 cells (2) 1.0 × 10^7^ (3) mice	(1) 7 days (2) NR	i.v.	(1) 5 mg/kg (2) 20 (3) 1 days	21 days	(1a) 7-fold (1b) 2-fold	(1) NR (2) NR
(1) Folic acid-liposome (2) ~190 nm [99]	(1) Celastrol (Cs) and Irinotecan (IR) (2) NR and (3a) 90% for both (3b) 28.5 ± 0.8% (Cs) and 14.7 ± 0.5% (Ir)	(1) MDA-MB-231 (2) 1.0 × 10^7^ (3) mice	(1) NR (2) 100 mm^3^	i.v.	(1) NR (2) 4 (3) 3 days	25 days	(1a) 3-fold (1b) 2-fold	(1) Yes (2) No
(1) Folic acid-lipid–polymer hybrid nanoparticles (NPs) (2) 279.9 ± 8.7 nm, [103]	(1) paclitaxel. (2) 20 mg/kg (3a) 91.16% ± 1.12% (3b) 27.36% ± 0.91%	(1) EMT6 (2) 2.0 × 10^7^ (3) mice	(1) NR (2) 100 mm^3^	Intratum-oral	(1) 20 mg/kg (2) 5 (3) 2 days	16 days	(1a) no significant reduction (1b) NR	(1) Yes (2) No
(1) Folic acid-Gold nanorods-liposome (2) 154 nm, [104]	(1) Doxorubicin (2) 2.5 mg/kg and (3a) 54.73 ± 2.13% (3b) NR	(1) 4T1 (2) 1.0 × 10^6^ (3) mice	(1) 10 days (2) 100 mm^3^	i.v.	(1) 2.5 mg/kg (2) NR (3) NR	15 days	(1a) 2.9-fold (1b) NR	(1) No (2) No
(1) Folic acid-PGG NPs (2) 131.96 ± 5.34 nm, [106]	(1) Docetaxel (2) 10 mg/kg and (3a) 67.83 ± 3.29(%) (3b) NR	(1) 4T1 (2) 1.0 × 10^5^ (3) mice	(1) NR (2) 100–200 mm^3^	i.v.	(1) 10 mg/kg (2) 4 (3) 7 days	28 days	(1a) 2.6-fold (1b) NR	(1) Yes (2) N0
(1) Folic acid-Quantum dot-PEG-PLGA polymersomes (2) 170.53 ± 1.21 nm, [107]	(1) Doxorubicin (2) 7 mg/kg and (3a) 54.26 ± 1.23% (3b) 10.82 ± 0.87	(1) 4T1 (2) 2.0 × 10^5^ (3) mice	(1) NR (2) 80–100 mm^3^	i.v.	(1) 7 mg/kg (2) 1 (3) 0 days	21 days	(1a) 5-fold (1b) NR	(1) Yes (2) No
(1) Folic acid-Dextran–PLGA polymersomes (2) 178.53 ± 2.5 nm, [109]	(1) Docetaxel (2) 10 mg/kg and (3a) 78.85 ± 3.81% % (3b) 9.32 ± 0.27	(1) 4T1 (2) 5.0 × 10^5^ (3) mice	(1) 7 days (2) 80–100 mm^3^	i.v.	(1) 10 mg/kg (2) 1 (3) 0 days	21 days	(1a) 4.5-fold (1b) NR	(1) Yes (2) No
(1) HER2 peptide- chitosan-liposome (2) 116.18 ± 1.73 nm, [114]	(1) Capecitabine (2) 10 mg/kg and (3a) 82.21 ± 0.62% (3b) NR	(1) MCF-7 (2) 1.5 × 10^5^ (3) mice	(1) 14 days (2) NR	i.v.	(1) 10 mg/kg (2) 9 (3) 2 days	21 days	(1a) 29-fold (1b) NR	(1) NR (2) NR
(1) HER2 peptide-liposome (2) ~80 nm [115]	(1) Doxorubicin (2) 3 mg/kg and (3a) NR (3b) > 98%	(1) MMTV/neu transgenic (2) NR (3) mice	(1) NR (2) ~150–200 mm^3^	i.v.	(1) 3 mg/kg (2) 5 (3) 2 days	25 days	(1a) 3.5-fold (1b) NR	(1) Yes (2) No
(1) HER2 (CD-340)-antibody-PLGA (2) 241 nm [119]	(1) Doxorubicin (2) 3 mg/kg and (3a) 88 ± 0.17% (3b) 8 ± 0.1	(1) SKBR-3 (2) 1.0 × 10^7^ (3) mice	(1) NR (2) 100–150 mm^3^	i.v.	(1) 3 mg/kg (2) 3 (3) 2 days	25 days	(1a) 2-fold (1b) NR	(1) No (2) No
(1) Estron ligand-pegylated liposome (2) ~120 nm [128]	(1) Paclitaxel (PTX), epirubicin (EPI) (2) 9.42 mg/kg (PTX), 6 mg/kg (EPI) and (3a) 90.23 ± 0.52 (EPI), 61.85 ± 0.56 (PTX) (3b) 5.07 ± 0.84 (EPI), 4.39 ± 0.67(PTX)	(1) MCF-7 (2) 2.0 × 10^6^ (3) mice	(1) NR (2) ~100–200 mm^3^	i.v.	(1) 9.42 mg/kg (PTX), 6 mg/kg(EPI) (2) 5 (3) 2 days	35 days	(1a) 6-fold (1b) NR	(1) Yes (2) No
(1) Hyaluronic acid-hybrid micelle (2) ~125 nm [133]	(1) Doxorubicin (2) 3 mg/kg and (3a) NR (3b) NR	(1) 4T1 (2) 2.5 × 10^5^ (3) mice	(1) NR (2) ~100 mm^3^	i.v.	(1) 3 mg/kg (2) 4 (3) 2 days	20 days	(1a) 4.8-fold (1b) 4.4 fold	(1) Yes (2) No
(1) Hyaluronic acid-p-CBA-Carbon dots (2) ~125 nm [134]	(1) Doxorubicin (2) 3 mg/kg and (3a) NR (3b) 18.13%	(1) 4T1 (2) 1.0 × 10^7^ (3) mice	(1) 6 days (2) ~50–100 mm^3^	i.v.	(1) 3 mg/kg (2) 5 (3) 2 days	21 days	(1a) 4-fold (1b) 2.5 fold	(1) Yes (2) No
(1) mPEG-HA/CSO-SS-Hex/SPION (2) ~100 nm [135]	(1) Gambogic acid (2) 6 mg/kg and (3a) 85.1% (3b) 23.7%	(1) 4T1 (2) NR (3) mice	(1) 15 days (2) 300–400 mm^3^	i.v.	(1) 6 mg/kg (2) 6 (3) 2 days	13 days	(1a) 10-fold (1b) 4- fold	(1) Yes (2) No
(1) Tf-PEG-NPs (2) 89 nm [140]	(1) Doxorubicin (DOX) Curcumin (CUR) (2) 50 mg/kg (DOX), 50 mg/kg(CUR) and (3a) 82.7 ± 4.1% (D-OX), 85.3 ± 3.2%(CUR) (3b) NR, 4.6 ± 0.8% (CUR)	(1) MCF-7 (2) 1.0 × 10^6^ (3) mice	(1) NR (2) NR	i.v.	(1) 50 mg/kg (2) 7 (3) 7 days	49 days	(1a) 6-fold (1b) NR	(1) No (2) No
(1) αvβ3 integrin RGD peptide-chitosan NPs (2) 200 nm [148]	(1) Raloxifene (2) 10 mg/kg and (3a) 50% (3b) NR	(1) 4T1 (2) 3.0 × 10^5^ (3) mice	(1) NR (2) NR	Oral	(1) 10 mg/kg (2) 4 (3) 1 days	13 days	(1a) 5-fold (1b) 2.8-fold	(1) No (2) No
(1) EGF and αvβ3 integrin peptide-liposome (2) 100 nm [149]	(1) Doxorubicin (2) 7.5 mg/kg and (3a) NR (3b) 18.13%	(1) D2. A1 (2) 5.0 × 10^5^ (3) mice	(1) NR (2) NR	i.v.	(1) 7.5 mg/kg (2) 3 (3) 1 days	49 days	(1a) NR (1b) NR,BLI signalling reduced to 1.33 fold	(1) NR (2) NR
(1) Biotinylated porous hexagonal ZnO nanodisc (2) 200 nm [151]	(1) Doxorubicin (2) 50 µg/mL and (3a) NR (3b) 63%	(1) EAC (2) 1.0 × 10^6^ (3) mice	(1) 12 days (2) 150 mm^3^	i.v.	(1) 50 µg/mL (2) NR (3) NR	28 days	(1a) 3.5-fold (1b) 5.3-fold	(1) NR (2) NR
(1) Biotinylated polymeric NPs (2) 105.8 ± 1.4 nm [154]	(1) Doxorubicin (DOX), Quercetin (QUT) (2) 5 mg/kg and (3a) 86% (DOX), 91% (QUT) (3b) 3.6% (DOX), 7.9% (QUT)	(1) MCF-7/ADR (2) 3.0 × 10^7^ (3) mice	(1) NR (2) 50 mm^3^	i.v.	(1) 5 mg/kg (2) 8 (3) 3 days	25 days	(1a) 1.95-fold (1b) 7-fold	(1) Yes (2) No
(1) LHRH-peptide conjugated dextran NPs (2) ~22 nm [158]	(1) Cisplatin (2) 4 mg/kg, 10 mg/kg and (3a) 85.5% (3b) 7.5%	(1) MCF-7 (2) 1.5 × 10^6^ (3) mice	(1) NR (2) 50 mm^3^	i.v.	(1) 4 mg/kg and 10 mg/kg (2) 3 (3) 4 days	20 days	(1a) 1.1-fold (4 mg/kg), 1.5-fold (10 mg/kg) (1b) NR	(1) Yes (2) No
(1) LHRH-peptide conjugated dextran NPs (2) ~22 nm [159]	(1) Cisplatin (2) 5 mg/kg, 10 mg/kg and (3a) 85.5% (3b) 7.5%	(1) 4T1 (2) 1.5 × 10^6^ (3) mice	(1) NR (2) 50 mm^3^	i.v.	(1) 5 mg/kg and 10 mg/kg (2) 3 (3) 4 days	20 days	(1a) 1.4-fold (5 mg/kg), 2.4-fold (10 mg/kg) (1b) 1.3-fold (5 mg/kg), 2.6-fold (10 mg/kg)	(1) Yes (2) No
(1) LHRH-peptide conjugated liposome (2) 103.3 ± 0.70 nm, [160]	(1) Mitoxantrone (2) 2.5 mg/kg, and (3a) 98.2% (3b) 9.5%	1) MCF-7 (2) 4.0 × 10^6^ (3) mice	(1) NR (2) 100 mm^3^	i.v.	(1) 2.5 mg/kg and (2) 3 (3) 7 days	21 days	(1a) 2-fold (1b) NR	(1) Yes (2) No

**Abbreviation and meaning:** EGF, epidermal growth factor; PMBN, poly (MPC-co-n-butyl methacrylate (BMA)-co-pnitrophenyloxycrabonyl poly (ethylene glycol) methacrylate) (MEONP); PLGA, poly (lactic-co-glycolic acid); PEG, poly (ethylene glycol); PGG, poly (l-g-glutamyl glutamine); p-CBA, 4-carboxybenzaladehyde; CSO, chitosan oligosaccharide; HEX, hexadecanol; SPION, superparamagnetic iron oxide nanoparticles; Tf, transferrin; LHRH, luteinizing hormone-releasing hormone.
